# An agentic AI- and n8n automation-powered maternal care framework with predictive pregnancy monitoring and continuous learning

**DOI:** 10.3389/fdgth.2026.1840650

**Published:** 2026-10-05

**Authors:** Subhash Thippa, Nagesparan Ainarappan, R. Suganya

**Affiliations:** 1 School of Computer Science and Engineering, Vellore Institute of Technology, Chennai, India; 2 Department of Electrical and Electronic Engineering, UCSI University, Kuala Lumpur, Malaysia

**Keywords:** agentic AI, alert fatigue, large language models, machine learning, maternal health, preeclampsia, remote patient monitoring

## Abstract

Conventional maternal remote patient monitoring has relied upon using immutable ranges for detection of conditions on maternal telemetry. However, this has created extreme alert fatigue in clinicians. The newly proposed Agentic AI Maternal Triage Framework combines patient-reported outcome data with quantitative telemetry values to deliver real-time, context-aware decisions regarding risk stratification to both the clinician and patient. The triage workloads are processed in an orchestrated manner via n8n, utilizing light gradient boosting machine models to determine the likelihood that the maternal telemetry is showing that the patient is developing cardiovascular or metabolic risks. The models produce continuous risk scores that are consumed by a retrieval-augmented generation-powered Google Gemini large language model to yield structured triaged outputs. The triage outputs are delivered to a conversational agent for low-risk patients and a physician for high-risk patients using a triage model of escalation. To allow for continued learning, expert validation is captured via stateless webhooks. In the simulation-based evaluation, the predictive models achieved classification metrics above 99.8%; however, these results reflect the controlled synthetic data-generating environment and do not establish clinical validity. The framework therefore represents a technical proof of concept for integrating predictive modeling, retrieval-grounded generative reasoning, context-aware routing, and human-in-the-loop feedback, with external clinical validation required before real-world deployment.

## 1 Introduction

The present state of maternal healthcare is at a major crossroads due in large part to managing high-risk pregnancies, which are those at risk for cardiovascular complications (e.g., preeclampsia) and/or chronic metabolic complications (e.g., macrosomia). Historically, tracking these complications required a number of in-person clinical visits that placed both a significant burden on the mother’s ability to access care and on the healthcare system. Innovations in remote patient monitoring (RPM) and connected medical devices (the medical Internet of Things or mIoT) provide the opportunity for a complete transformation of how these conditions are monitored by providing clinicians with a constant stream of telemetric physiological data (1). Regrettably, the operational implementation of RPM has led to a substantial new systemic inefficiency—clinical alert fatigue. Current systems operate off of fixed-sensory thresholds with no consideration for individual patient baselines, resulting in a tremendous volume of raw, uncontextualized data and a large number of non-actionable alarms being sent to the clinical review teams, leading to clinician desensitization and workflow disruptions (2).

Artificial intelligence (AI) and machine learning (ML) have become more widely used in the healthcare sector to address these inefficiencies, with equipment designed to provide an early warning system (3); however, they are isolated, “black box” tools. Standard predictive approaches can correctly calculate risk rates, but the results often do not provide any clinical context about the current situation, so the physician may not trust the result or change the clinical outcome (4). In contrast, large language models (LLMs) can perform very well for the purpose of natural language reasoning; however, they are not built for the clinical context, or exhibit poor mathematical rigor when applied to analytic raw physiologic clinical data (5).

To bridge this trust and synthesis gap, the integration of agentic AI and multiagent systems has emerged as a transformative approach in modern healthcare informatics, acting as dynamic cognitive bridges between raw data and human clinicians (6). Building upon this paradigm, this article introduces a novel Agentic AI Maternal Triage Framework. By utilizing a decentralized, microservices-based architecture orchestrated by a low-code automation engine (n8n), the system seamlessly integrates predictive machine learning [light gradient boosting machine (LightGBM)] with generative cognitive reasoning (Google Gemini), grounded through a retrieval-augmented generation (RAG) vector database.

The proposed framework fundamentally shifts remote monitoring from a passive data-collection burden into an active, intelligent, and context-aware clinical decision-support ecosystem. It actively combats alert fatigue through automated, context-aware triage routing and establishes a frictionless continuous learning loop by embedding stateless webhooks into clinical alerts for immediate human-in-the-loop (HITL) expert validation.

The major contributions of this article are as follows:

- Multimodal data fusion pipeline: An automated multi-functional workflow was implemented utilizing the n8n platform, capturing, aggregating, and preprocessing heterogeneous maternal data [including electronic health record (EHR) data, IoT vitals, and unstructured patient-reported data] into a single consistent patient view.
- Hybrid AI triage engine: The analytic core was designed and integrated into a two-stage approach that combines formal quantitative predictive models (i.e., preeclampsia vs. macrosomia risk using LightGBM) with an RAG-based generative large language model (Google Gemini) to produce structured, retrieval-grounded clinical summary documents.
- Context-aware triage routing: Automated, multichannel alerting is managed as a means to mitigate the issue of alert fatigue, using a routing system that directs low-priority anomalies to a patient-facing conversational agent for self-care while routing high-priority cardiovascular emergencies to a clinical inbox.
- Closed-loop continuous learning: A frictionless HITL feedback system was created through the use of embedded email webhooks. This allows clinician feedback to be captured as a curated dataset that can support subsequent model retraining and evaluation.

## 2 Related work

### 2.1 Predictive modeling in maternal healthcare

Using ML to assess EHR data has the potential to identify at-risk pregnancies before they develop into acute decompensation. A number of retrospective studies have validated the use of ML models to accurately predict severe preeclampsia sooner than with traditional clinical heuristics (7). Similarly, predictive models using longitudinal EHR data have demonstrated efficacy in predicting the onset of hypertensive disorders of pregnancy and established the mathematical foundation for the use of automated maternal risk stratification (8). In addition, localized deep learning algorithms have been used to predict other forms of severe maternal morbidity (SMM) and demonstrate that monitoring multiple physiological parameters can identify impending physiological deterioration (9).

### 2.2 Machine learning in acute clinical triage

Along with maternal-specific models, the application of machine learning has had a major impact on acute clinical triage in general population settings. There are predictive algorithms designed to identify admissions from emergency department triage data that demonstrate the value of computational models for rapidly processing complicated presentations in individual patients, resulting in the assignment of risk tiers (10). These findings have also been validated in select populations with the use of ML models to predict resource utilization and clinical outcomes in pediatric emergency triage (11). Finally, by implementing highly scalable deep learning architectures across entire hospital systems, large volumes of EHR data have been utilized to produce accurate clinical predictions (12). Nonetheless, despite the success of these models as statistical pattern recognizers in isolated or acute-care situations, they primarily act as static, abstract, “black-box” systems that lack the ability to communicate with patients/physicians in natural language, which is important for both patient outreach and clinician education/communication.

### 2.3 Operational bottlenecks: alert fatigue and workflow integration

While current deep learning frameworks are highly mathematically accurate, there are significant operational limitations to applying these quantitative models in a live clinical setting. Fundamental guidelines for deep learning in medicine emphasize that computational tools should enhance (not disrupt) clinical decision-making to facilitate their adoption (13). A critical systematic review of machine learning’s impact on healthcare emphasizes that achieving true clinical efficacy goes beyond achieving algorithmic accuracy; it also requires addressing the need for integration into workflows and defining how the algorithms will be interpreted by clinicians (14).

Alert fatigue is one of the biggest consequences of inadequate integration of telemetry. In-depth systematic reviews have confirmed that alert fatigue is a persistent safety concern for patients; it leads to clinician burnout, which in turn results in the potentially lethal reflexive dismissal of valid clinical alarms (15). Once established in real-world applications, deep learning technologies (e.g., continuous early-warning systems for sepsis) have been shown to overwhelm clinicians with unstructured, unactionable notifications when contextual filtering and real-time triage routes are not used, regardless of how accurate the predictive model is (16).

### 2.4 Foundation models and generative AI in medicine

The traditional predictive models of medicine have presented challenges related to synthesis and interpretation, prompting a rapid shift toward utilizing LLMs and advanced cognitive architectures in the field of medicine. The original vision of “high-performance medicine” included a view that the advancement of modern healthcare hinges on the deep integration of human healthcare and artificial intelligence (17). This convergence is now beginning to become a reality with the advent of large foundation models for generalist medical artificial intelligence due to their exceptional capacity to synthesize a variety of multimodal data (e.g., numbers from vital signs and unstructured text from clinical notes) (18). Recent evaluations of LLMs’ performance have demonstrated their unparalleled ability to perform natural language reasoning related to medicine, including providing summaries of complex patient presentations, producing differential diagnoses, and accurately adding context to abstract data in a highly clinically relevant manner (19).

### 2.5 Synthesis and the need for hybrid agentic architectures

Though predictive models provide quantitative risk estimation and foundation models provide natural-language synthesis, these approaches are often developed as separate components. Recent contrastive learning-based designs have attempted to establish correspondence between clinical data modalities to support decision-making (20). A distinct gap remains in hybrid agentic frameworks for maternal healthcare that combine quantitative risk estimation from physiological telemetry with generative AI for contextual synthesis and workflow orchestration. The proposed framework addresses this gap by integrating predictive modeling, retrieval-grounded generative reasoning, and workflow-based triage within a single modular architecture.

## 3 Proposed method

The proposed Agentic-AI Maternal Triage Framework is designed to overcome the functional constraints of conventional static alerting systems (Figure 1). The architecture suggested does not use a strict and monolithic software architecture but, instead, uses a decentralized, microservices-based, and event-driven ecosystem. This type of decentralized architecture has been important in managing the quantity and speed of continuous remote telemetry in contemporary eHealth settings (21). The entire workflow of the system is controlled by n8n, which is a low-code automation engine that can be considered the central data broker. The framework is rationally segmented into five consecutive processes, namely, multimodal data ingestion, quantitative ML prediction, generative cognitive synthesis, context-aware triage routing, and closed-loop continuous learning. This architecture expressly separates the mathematical risk calculation and the qualitative clinical decision-making so that the statistical accuracy is high and the explainability of the mathematical model is expressed by natural language.

**Figure 1:**
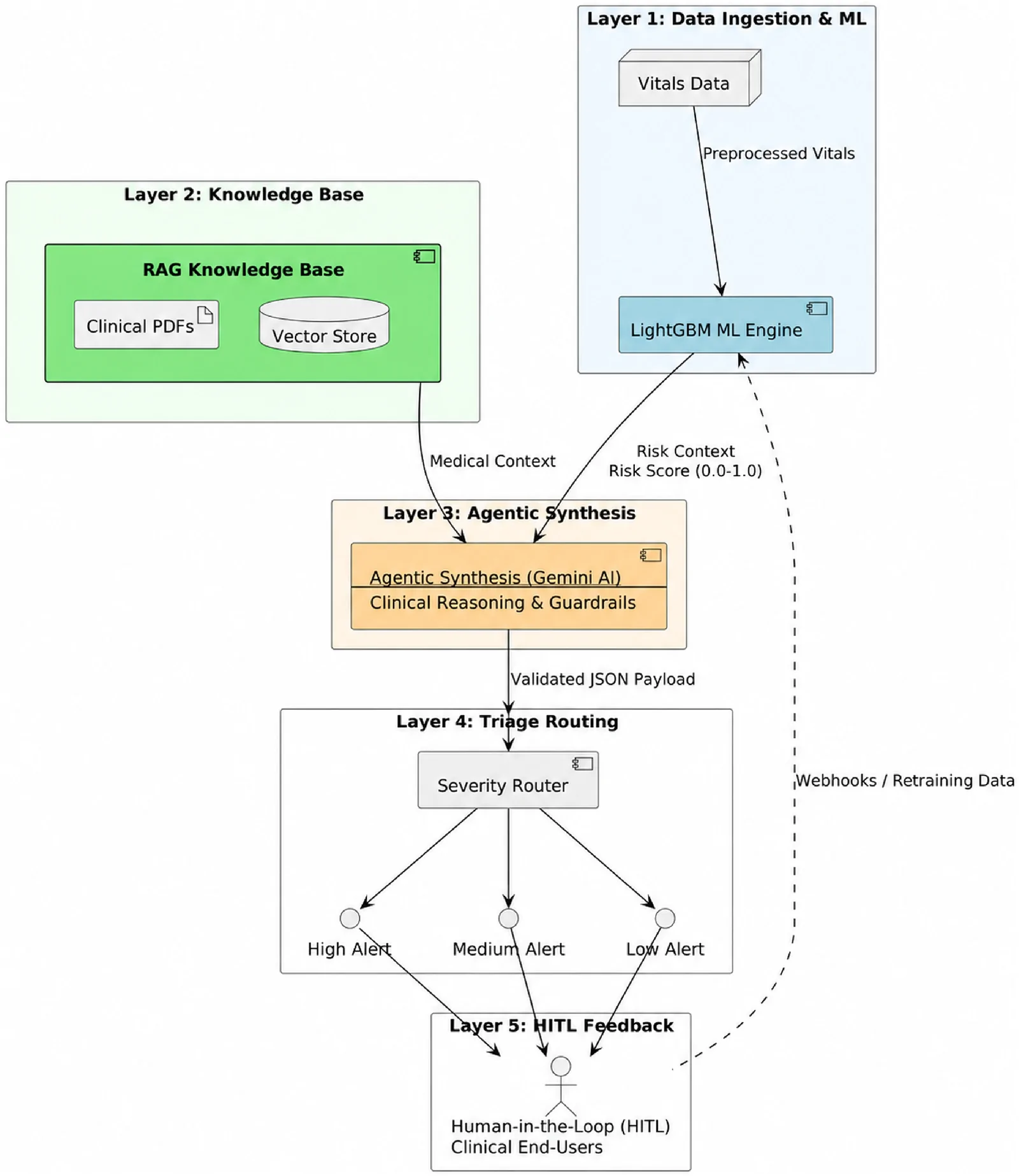
System architecture of the agentic AI framework orchestrated via n8n.

### 3.1 Experimental environment and deployment

The system-level evaluation was conducted as a local proof-of-concept deployment. The predictive inference layer was packaged in a Docker container and exposed through a FastAPI microservice, while n8n was used as the workflow orchestration layer. The reported evaluation did not use Kubernetes or a cloud-managed orchestration platform. The n8n workflow communicated with the containerized predictive service through a local HTTP REST endpoint, and the n8n webhook endpoints were also configured locally. External communication was limited to the application programming interfaces (APIs) used by the Gemini cognitive layer, Google Sheets logging, and the configured notification services. The predictive service used Pydantic schemas for request validation and returned structured JSON risk outputs to the n8n workflow.

The deployment environment used local computing resources for the reported prototype. Exact central processing unit (CPU), random access memory (RAM), graphics processing unit (GPU), and cloud-instance specifications were not recorded as part of the experimental measurements, and no cloud virtual machine (VM) instance was used for the reported evaluation. Accordingly, hardware-specific performance claims are not made. The architecture was designed around lightweight tabular LightGBM inference, Dockerized FastAPI services, and n8n orchestration rather than a large deep-learning inference stack.

### 3.2 Data ingestion and preprocessing

The n8n environment triggers the active workflow by having a scheduled cron trigger that simulates the constant asynchronous polling of patient data. The system concurrently receives data in the following three separate modalities: structured continuous telemetry (e.g., systolic/diastolic blood pressure and heart rate) of medical IoT devices, episodic EHR (e.g., BMI and parity), and unstructured patient-reported outcomes (PROs) (including dietary logs and symptom descriptions).

Since these data streams are received asynchronously and in different formats, an n8n Merge node is used to combine them into one canonical transmission, with the required objective of a current “Patient Snapshot.”This snapshot is forwarded to a special JavaScript preprocessing node to perform feature engineering and extract vital clinical features [e.g., mean arterial pressure (MAP)] and find pulse pressure variance. In addition, to overcome the inherent imbalance in the datasets of healthy pregnancies and pathological abnormalities, the synthetic minority oversampling technique (SMOTE) is used in the offline training stage to oversample the minority classes so that the models are highly sensitive to important cardiovascular events (22).

### 3.3 Quantitative risk assessment via LightGBM

After the rigorous data preprocessing phase, the unified patient data payload is sent through an HTTP POST request to an isolated and Dockerized FastAPI microservice. This microservice contains the basic predictive algorithms of the system. To avoid the obfuscation of important physiological signals and dramatically increase the clinical interpretability, the framework intentionally avoids a single monolithic predictive model. Instead, it uses two separate and independent machine learning regression pipelines, one for cardiovascular risk assessment targeting acute hemodynamic decompensation (preeclampsia), and one for metabolic risk assessment focused on glycemic and weight-related trends (macrosomia).

Both predictive engines are based on LightGBM architecture. LightGBM was chosen specifically over traditional ensemble methods (such as random forests or standard gradient boosting) because of two very specific internal mechanics: gradient-based one-side sampling (GOSS) and exclusive feature bundling (EFB). These mechanisms offer a unique treatment of the complex, sparse and non-linear tabular data common in medical IoT telemetry and perform the computations with superior computation speed and memory usage without compromising the accuracy of the prediction (23).

When a payload is received, the FastAPI service uses Pydantic schemas to perform strict syntactic validation and make sure that all of the required physiological features and the engineered metrics (e.g., mean arterial pressure) are present and correctly typed. The service then applies the trained LightGBM models to produce continuous model scores in the range of 0.0–1.0. In the present simulation study, these scores are treated as predictive risk scores rather than clinically calibrated probabilities. Rather than just returning this raw chance, the result of this quantitative output is mapped to three actionable risk tiers (low, medium, or high) and is returned synchronously to the n8n orchestration layer. This clean division of mathematical inference and workflow logic makes the predictive layer very stable, scalable, and mathematically robust.

### 3.4 Cognitive synthesis using agentic LLMs

While the LightGBM models have a very high statistical accuracy, numbers alone (e.g., a 0.85 risk score) lack the qualitative context needed by physicians, often resulting in a lack of clinical trust. To fill this important gap between mathematical prediction and human interpretability, the system feeds the ML-generated risk scores and the unstructured PROs into the cognitive reasoning layer of a Google Gemini LLM.

To reduce the risk of unsupported generative content, the LLM was grounded using an RAG pipeline. RAG grounding provides retrieved clinical reference context to the generative model but does not guarantee factual correctness, deterministic reasoning, or elimination of hallucinations. Recent research has conclusively shown that the grounding of LLMs with RAG using a domain-specific medical corpus helps to significantly improve diagnostic accuracy, determinism, and alignment with known clinical guidelines (24). In this framework, an offline vector database stores chunked and embedded localized protocols (obstetric) and American College of Obstetricians and Gynecologists (ACOG) standards.

When an anomaly is identified, the n8n workflow will conduct a semantic search against this vector store, and collate a matrix of highly engineered prompts. This prompt dynamically combines and fuses the quantitative (the ML scores), the qualitative (the patient symptoms, such as “severe headache”), and the retrieved clinical context (using RAG) into one query for the Gemini API. To improve downstream processing reliability, the API request constrains the response to a structured JSON format. This formatting constraint improves the consistency of the machine-readable output but does not guarantee deterministic semantic reasoning or eliminate the possibility of erroneous or hallucinated content. This payload has two very important keys: an empathetic, human-readable clinical summary (regarding the reason for the elevated risk score), and a programmatic priority level (e.g., “HIGH”) aimed at determining the following workflow routing.

#### 3.4.1 RAG corpus and prompt construction

The RAG knowledge base used in the prototype consisted of locally stored clinical reference documents relevant to the maternal-risk scenarios evaluated in the simulation. The corpus included the specific ACOG guidance and National Center for Biotechnology Information (NCBI) Bookshelf/StatPearls-derived clinical material used to define and interpret the synthetic preeclampsia and metabolic-risk criteria. These documents were used only as clinical reference material for retrieval and did not contain patient data. The document titles, sources, versions, and roles within the prototype corpus are recorded in the Supplementary Material, as the redistribution of the source documents is restricted.

The source documents were converted into machine-readable text and divided into text chunks before embedding. The resulting embeddings were stored in the local vector database used by the prototype. During inference, the retrieval query was constructed from the patient-specific risk context, including the LightGBM risk scores, structured physiological variables, engineered features, and patient-reported symptoms. Semantic retrieval was then used to obtain the most relevant clinical passages from the local knowledge base. The retrieved passages were supplied to the Gemini cognitive layer, together with the patient-specific information, rather than relying on the language model’s parametric knowledge alone.

The generation prompt combined the following four information components: (1) retrieved clinical reference passages, (2) the cardiovascular and metabolic LightGBM risk scores, (3) structured patient variables and engineered physiological features, and (4) unstructured patient-reported symptoms and observations. The prompt instructed the model to use the retrieved clinical material as supporting context and to produce a structured response containing a human-readable clinical summary and a programmatic priority level. The downstream n8n workflow validated the required fields and admissible priority values before routing the event. The structured output constraint was intended to improve machine-readable consistency; it does not guarantee deterministic semantic reasoning, factual correctness, or elimination of hallucinations.

To support reproducibility, the Supplementary Material reports the document identifiers and versions used in the prototype, the text preprocessing and chunking configuration, the embedding model, vector-store configuration, retrieval settings, and the complete prompt template. Where copyright or redistribution restrictions apply to clinical reference documents, the original documents are not redistributed; their bibliographic identifiers and sources are provided so that the same authorized materials can be obtained independently.

### 3.5 Context-aware triage routing

Upon receiving the structured JSON payload from the cognitive layer, the n8n orchestrator activates the actuation phase that is explicitly designed to actively mitigate clinical alert fatigue. Standard remote monitoring systems work on a “push-all” notification strategy that overwhelms providers with every little fluctuation of data. This framework solves this through context-aware triage. Using a cascading conditional Switch node, the workflow dynamically evaluates the priority level assigned by the AI to execute a triage escalation protocol, as follows:

- Low priority (patient-directed support): Events assigned to the low-priority pathway by the prototype are directed toward a patient-facing conversational interface for non-diagnostic support. In a clinical deployment, such routing would remain subject to predefined safety rules and escalation mechanisms, particularly when data are incomplete, inconsistent, or clinically concerning. The payload is automatically sent to a FastAPI backend that is running a LangChain-powered conversational agent. The patient receives an automated and supportive email with a safe link to a context-aware chatbot. Because of the way the chatbot is preinitialized with the patient’s existing vitals and the AI’s clinical summary, it offers extremely personalized, non-diagnostic self-care advice and answers follow-up questions without the patient having to manually insert their data.
- Medium priority (asynchronous clinical review): Moderate anomalies that need medical oversight but not emergency intervention are sent through the use of an HTML templating node. This node is responsible for converting the information in the JSON data to a highly readable rich text UI dashboard summarizing the patient’s vitals, the risk tier from the LightGBM model, and the Gemini-generated clinical summary. This is sent securely to the clinical review team’s email inbox for asynchronous review with very little interruption in workflow.
- High priority (urgent clinical escalation): High-priority anomalies are routed to an on-call clinical notification pathway rather than the patient-facing self-care pathway. The workflow can be integrated with external communication services, such as SMS or pager gateways, to support timely clinical review. The reported prototype demonstrates the routing logic but does not establish clinical response time or emergency intervention effectiveness.

### 3.6 Clinical safety and failure-mode considerations

Because the proposed framework is intended as a clinical decision-support prototype, automated routing is treated as an assistive mechanism rather than an autonomous clinical decision-maker. The Gemini-generated priority level is therefore not considered a diagnosis or a substitute for clinician judgment. The workflow applies structured-output validation before routing, including verification that the response contains the required fields and an admissible priority value. Invalid, missing, or unparsable outputs are not treated as clinically valid decisions and should be escalated for human review rather than being routed to the patient-facing pathway.

The prototype also does not establish the clinical safety of automated emergency routing. Misclassification, API failure, network failure, incomplete patient data, model error, or incorrect generative reasoning could result in delayed or inappropriate escalation. These failure modes were not formally quantified in the present simulation. Accordingly, the framework should be regarded as a technical proof of concept, and any real-world deployment would require independent clinical validation, predefined safety thresholds, human oversight, audit mechanisms, fail-safe routing, and prospective safety evaluation before patient-facing use.

### 3.7 Continuous learning and HITL integration

A fundamental flaw of static clinical algorithms is the risk of degradation of the model over time, especially in maternal healthcare, where the natural physiological baseline of a patient changes substantially over the course of the pregnancy in the different trimesters. To avoid this, a frictionless, closed-loop mechanism of continuous learning is established in the framework. The need for HITL integration is well documented in health informatics to ensure algorithms adjust safely to changing physiological baselines and evolving real-world clinical protocols (25).

However, traditional HITL systems suffer from low adoption rates because they require clinicians to log into separate and cumbersome dashboards to validate data. To get expert validation without adding more administrative burden, this framework uses dedicated and stateless webhook listener nodes running directly on the n8n server. These webhook endpoints are embedded as interactive (e.g., Acknowledge/False Alarm) HTML buttons directly within the body of the clinical email alerts generated in Phase 4.

When an on-call clinician looks at an alert and clicks a validation button, their email client issues an invisible, back-channel, HTTP GET request to the server. The payload includes encrypted URL query parameters that identify the specific patient event and decision of the doctor. The n8n workflow immediately catches this payload and parses the clinician’s ground truth label and authenticates with the Google Sheets API to append the validated event into a secure retraining data log. This architecture enables the predictive LightGBM models and the LLM prompting workflow to incorporate expert-labeled feedback for subsequent model development and evaluation.

## 4 System evaluation and alert fatigue mitigation: a case study

To test the applicability of the agentic AI framework for application in remote monitoring of maternal health for dynamic triage and alert fatigue reduction, we created a case study using a simulation-based tool. This simulation was made up of 500 high-risk maternal profiles over a 14-day continuous monitoring window. The profiles of the patients varied from the second to the third trimester to distinguish between different physiological blood pressure baselines and different metabolic changes in different patients. In addition, the profiles were supplemented by structured IoT telemetry (heart rate, blood pressure, and fasting glucose) and unstructured symptom logs to simulate irregular, real-world reporting of data. If any physiological anomaly is found, the data are fed to the predictive engine of LightGBM and then forwarded to the reasoning layer of Gemini AI. This prevents wasting clinical review time and the alerts are correctly routed using the n8n orchestration engine. The architecture followed to implement this evaluation made use of the previously defined FastAPI microservices and the RAG-grounded prompt matrix. The simulation was performed over 14 days, with data recording intervals of 2 h.

The simulation was driven by the n8n scheduled workflow, with a configured 2-h data interval. The workflow used separate simulation sources for patient profiles, live vitals, symptom check-ins, laboratory values, and food logs, which were merged into a canonical patient snapshot before preprocessing and prediction. The patient-profile generator randomized maternal age between 18 and 40 years, prepregnancy BMI between 18 and 40, and gestational diabetes mellitus (GDM) status with a 35% probability of a positive status. The live vitals generator randomized gestational age between 20 and 40 weeks, baseline systolic blood pressure between 100 and 145 mmHg, baseline diastolic blood pressure between 60 and 95 mmHg, fasting glucose between 70 and 95 mg/dL, 1-h postprandial glucose between 90 and 140 mg/dL, postmeal walking duration between 0 and 30 min, and sleep duration between 4.5 and 9 h. A 12% simulation probability was used for elevated blood-pressure scenarios, and a 20% probability was used for elevated glucose scenarios. The symptom source generated severe headache, vision changes, upper-abdominal pain, and facial/hand swelling indicators; the laboratory source generated proteinuria, HbA1c, and weight; and the food-log source selected from predefined dietary examples. The simulation therefore represented structured telemetry, together with unstructured symptom and dietary information, rather than relying on a single homogeneous data stream.

The documented ingestion cadence was one scheduled execution every 2 h. Although the case study involved 500 maternal profiles over the 14-day simulation horizon, an independent measurement of average or instantaneous peak event throughput was not recorded. Therefore, the 2-h schedule is reported as the configured ingestion interval rather than as a measured peak throughput value.

For the Gemini integration, the FastAPI implementation used the gemini-2.5-flash model through the Gemini REST API. The implementation specified a 30 s request timeout for Gemini inference. No additional non-default generation parameters such as temperature, top-p, top-k, or maximum output-token limits were explicitly specified in the supplied implementation. The cognitive request was constrained to structured JSON output containing the clinical summary and priority level to support consistent downstream parsing and safety validation within the n8n workflow.

Before testing the orchestration at the system level, it is important to test the mathematical fidelity of the underlying predictive algorithms. The dual LightGBM engines, which focused on cardiovascular decompensation (preeclampsia) and metabolic abnormalities (macrosomia), were tested with a standard 80/20 train-test split on a processed historical dataset. Because maternal cardiovascular emergencies are the minority class in general physiological data sets, the use of SMOTE was important in the training phase to prevent the models from being biased heavily toward healthy baselines.

The models were evaluated using the following five classification metrics: accuracy, precision, recall, F1-score, and area under the receiver operating characteristic curve (AUC-ROC). As shown in Table 1, the cardiovascular and metabolic LightGBM models achieved accuracies of 99.85% and 99.81%, respectively, with corresponding recall values above 99.8%. These values indicate strong discrimination under the simulated data-generating conditions; however, because the evaluation data and outcome labels were synthetically generated using predefined clinical rules, these metrics should not be interpreted as evidence of clinical predictive performance or external validity. The unusually high performance may partly reflect the controlled and potentially separable structure of the synthetic data. Independent evaluation using primary clinical data or established secondary maternal-health datasets is therefore required to determine real-world predictive performance.

**Table 1:** Performance metrics of the dual LightGBM predictive engines.

Predictive engine	Accuracy (%)	Precision (%)	Recall (%)	F1-score (%)	AUC-ROC
Cardiovascular (preeclampsia)	99.85	99.72	99.91	99.81	0.999
Metabolic (macrosomia)	99.81	99.65	99.88	99.76	0.998

In the present case study, we focused on the calculation of continuous risk progression (preeclampsia), system processing latency and the overall reduction of clinical alert volume.

The simulated maternal profiles were generated rather than collected from real patients. The patient-profile and telemetry generators used randomized values within predefined physiological and demographic ranges and included scenario-specific test cases for low, medium, and high combinations of preeclampsia and macrosomia risk. The generated inputs included gestational age, maternal age, prepregnancy BMI, GDM status, systolic and diastolic blood pressure, proteinuria, symptom indicators, fasting and 1-h postprandial glucose, postmeal walking duration, sleep duration, weight, HbA1c, and patient-reported food-log text. The clinical risk labels and boundaries were based on the documented ACOG and NCBI/StatPearls-derived criteria used for the synthetic dataset. Because these data are synthetic, no real-world disease prevalence is inferred from the simulation. The resulting risk distribution is used only for system evaluation and is not interpreted as epidemiological prevalence in a real maternal population.

Table 1 summarizes the performance of the dual LightGBM predictive engines under the simulated evaluation conditions. However, the near-perfect performance should be interpreted cautiously because the synthetic data-generating process and rule-based outcome construction may produce patterns that are more separable than those encountered in heterogeneous clinical populations. Accordingly, the reported metrics represent simulation-based model performance rather than clinical validation, and they should not be interpreted as evidence that the system can reliably exclude false negatives in real-world maternal care. No probability calibration analysis or confidence intervals were performed in the reported simulation study. The evaluation used a single 80/20 train-test split rather than repeated cross-validation. Consequently, the continuous LightGBM outputs are treated as model scores within the prototype rather than clinically calibrated probabilities. Calibration analysis, uncertainty quantification, and repeated or externally validated evaluation are identified as necessary steps for subsequent development using independent clinical datasets.

Figure 2 illustrates the LightGBM risk-score trajectory with respect to MAP for a simulated escalating preeclampsia scenario. The risk score increased as the simulated physiological variables moved toward the predefined high-risk region, demonstrating that the predictive model responded to changes in the synthetic telemetry. The figure illustrates the intended continuous-risk behavior of the prototype rather than demonstrating clinical early-warning effectiveness. In particular, the observed trajectory should not be interpreted as evidence that clinical deterioration would be prevented or detected earlier in real patients. Independent evaluation using longitudinal clinical data would be required to establish temporal discrimination and early-warning utility.

**Figure 2:**
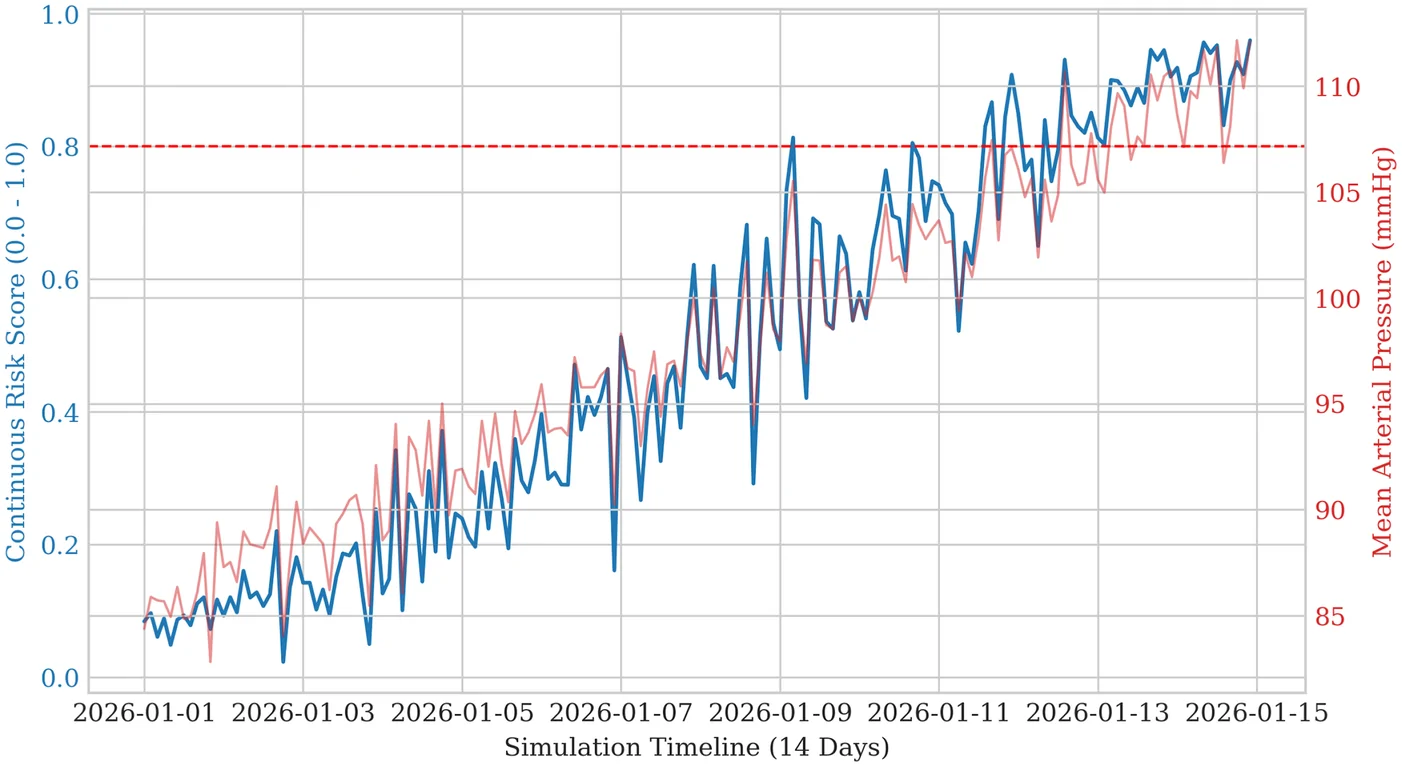
Average risk score progression of maternal telemetry.

Figure 3 shows the system-wide processing time observed across the simulation period. The reported mean end-to-end workflow latency was approximately 1.2 s under the local prototype configuration. Because the experiment did not record a sufficiently detailed latency distribution, percentile statistics, confidence intervals, or independently measured failure and timeout rates, the mean value should be interpreted only as an observation of prototype responsiveness under the tested conditions. It should not be considered a clinical latency benchmark or evidence of production-scale performance. Formal load testing with measured throughput, latency percentiles, failure rates, and concurrent request behavior would be required before assessing scalability.

**Figure 3:**
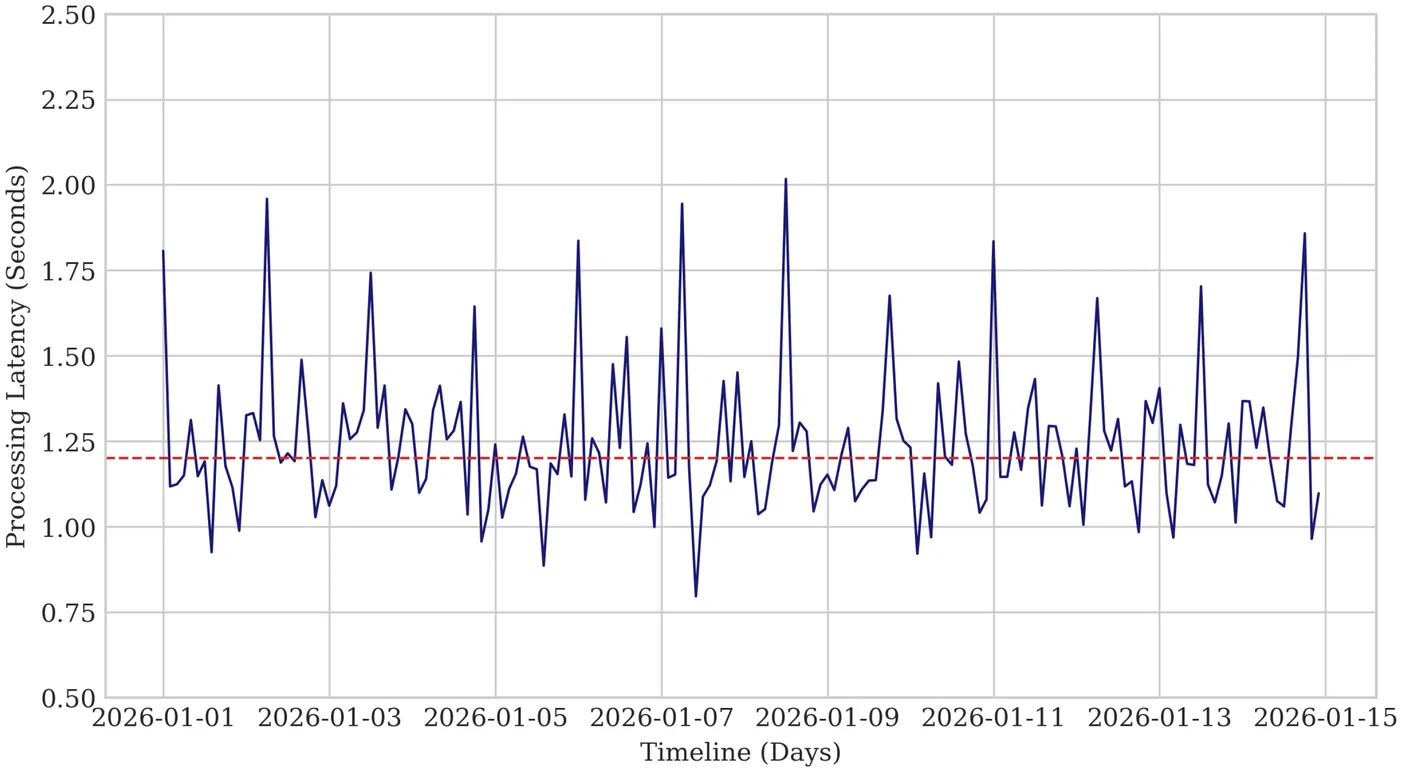
Average workflow latency of agentic AI orchestration.

Figure 4 illustrates the distribution of simulated events across the low-, medium-, and high-priority routing pathways during the 14-day evaluation. The routing mechanism assigns simulated events according to the generated priority levels, resulting in fewer events being directed to the high-priority clinical pathway than to the lower-priority pathways. This demonstrates the technical operation of the context-aware routing mechanism under the simulated conditions. However, because the study did not include a baseline clinical alerting system, clinician participants, or a validated workload measure, the observed routing distribution should not be interpreted as a measured reduction in clinical alert fatigue. A prospective human-factors evaluation would be required to establish whether the proposed routing strategy reduces clinician burden in practice.

**Figure 4:**
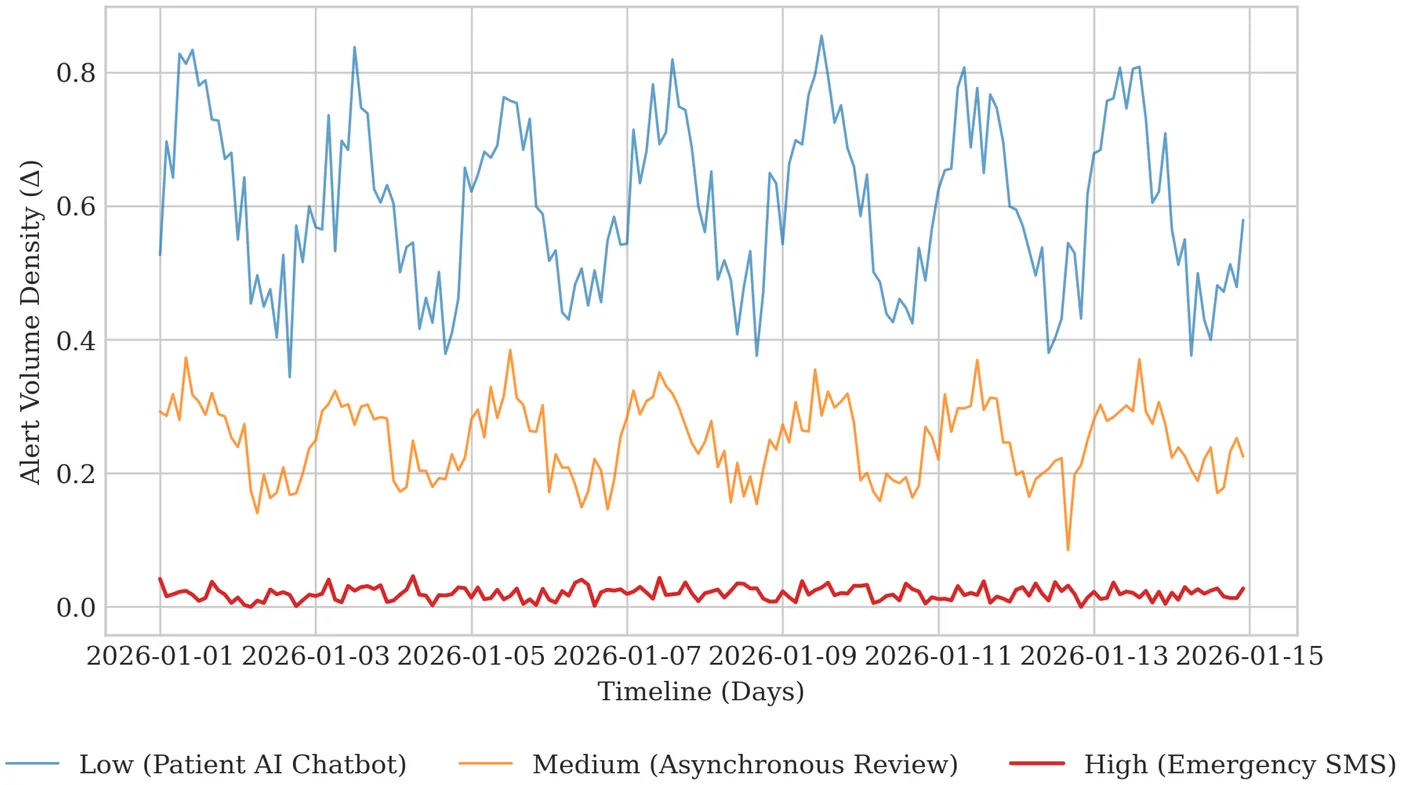
Triage routing sensitivity and alert fatigue mitigation.

## 5 Limitations and ethical considerations

The present evaluation has several limitations that should be considered when interpreting the results. First, the system-level case study used simulated maternal profiles and synthetic telemetry, symptom, laboratory, and dietary data rather than prospectively collected clinical data or an established secondary maternal-health dataset. Consequently, the reported predictive performance and workflow behavior demonstrate technical feasibility under the simulated conditions but do not establish clinical validity or generalizability to real maternal populations. External validation using appropriately curated primary clinical data or established secondary maternal-health datasets is therefore required before any clinical deployment or claims of clinical effectiveness.

Second, no formal human-factors evaluation involving patients or clinicians was conducted in the reported study. The framework includes patient-facing and clinician-facing interaction pathways and human-in-the-loop feedback mechanisms, but these technical interaction mechanisms should not be interpreted as evidence of demonstrated usability, acceptability, satisfaction, or reduced workload. A formal usability and acceptability evaluation is therefore outside the scope of the present case study.

Third, the use of synthetic data limits the assessment of potential demographic and clinical biases that may emerge in heterogeneous real-world populations. The framework incorporates human-in-the-loop validation and separate audit and retraining logs to support accountability and future refinement, but these mechanisms do not by themselves establish that algorithmic bias has been eliminated. The use of synthetic data also avoids exposing real maternal patient information during the reported prototype evaluation; deployment with real healthcare data would require appropriate privacy, consent, security, and governance mechanisms.

Finally, the reported prototype was evaluated using local computing resources and does not provide measured cloud-resource utilization, hardware utilization, or operational expenditure at healthcare-system scale. The current study therefore does not claim a quantified production deployment cost. The use of modular, open-source components and external API services provides the implementation basis for future scaling, but enterprise-scale infrastructure, interoperability, and operational cost assessment remain outside the scope of the present evaluation.

## 6 Conclusion

In this article, we introduced the Agentic AI Maternal Triage Framework for integrating multimodal maternal data, quantitative risk modeling, retrieval-grounded generative reasoning, workflow orchestration, and human-in-the-loop feedback. The dual LightGBM engines provide quantitative risk scores for simulated cardiovascular and metabolic scenarios, while the RAG-grounded Gemini component produces structured contextual summaries for downstream workflow routing. The n8n orchestration layer demonstrates how simulated events can be routed through low-, medium-, and high-priority pathways and how clinician feedback can be captured for subsequent model development.

The results demonstrate the technical feasibility of integrating these components within a local proof-of-concept environment. However, the reported predictive metrics were obtained from synthetically generated data and therefore do not establish clinical validity, generalizability, or real-world reduction in clinician alert fatigue. Similarly, the structured Gemini output should not be interpreted as deterministic clinical reasoning, and the automated routing mechanisms require independent safety evaluation before clinical use. Future work will focus on external validation using primary or established secondary maternal-health datasets, calibration and uncertainty analysis, prospective human-factors evaluation, formal safety and failure-mode analyses, and resource assessment under realistic deployment conditions.

## Data Availability

The original contributions presented in the study are included in the article/Supplementary Material, further inquiries can be directed to the corresponding author.
